# Editorial: From Peptide and Protein Toxins to Ion Channel Structure/Function and Drug Design

**DOI:** 10.3389/fphar.2020.548366

**Published:** 2020-09-25

**Authors:** Annette Nicke, Chris Ulens, Jean-Francois Rolland, Victor I. Tsetlin

**Affiliations:** ^1^ Walther Straub Institute of Pharmacology and Toxicology, Faculty of Medicine, Ludwig-Maximilians-Universität München, Munich, Germany; ^2^ Laboratory of Structural Neurobiology, Department of Cellular and Molecular Medicine, KU Leuven, Leuven, Belgium; ^3^ Axxam SpA, Milan, Italy; ^4^ Department of Molecular Neuroimmune Signaling, Shemyakin-Ovchinnikov Institute of Bioorganic Chemistry, Russian Academy of Sciences, Moscow, Russia

**Keywords:** α-conotoxins, Three-finger Ly6 proteins, nicotinic receptors, Cys-loop receptors, Voltage-gated ion channels, receptor complexes, drug design

From the very beginning of receptor research, animal toxins have been crucial for the identification of ion channels and their subtype differentiation, biochemical investigation, and cloning. Today, they also serve as invaluable tools for the detailed structural characterization of receptors and might even have drug potential or serve as templates for drug development.

In this broadly planned topic, we included recent research and reviews on both three-fingered proteins with homology to neurotoxins from snakes and much smaller peptide toxins from cone snails, spiders, and scorpions. In line with the activity of the respective toxins, the articles describe their use as tools for research or potential therapeutic applications on ligand-gated nicotinic acetylcholine receptors (nAChRs), voltage-gated Na^+^ and K^+^ channels, and, last but not least, the effects of chlorotoxin fragments on cell migration via an unidentified target.

Two papers focus on endogenous prototoxins of the Ly6 family, a diverse family of three-fingered proteins with multiple physiological functions in mammals. J. M. Miwa who contributed to the discovery of this protein family together with her co-authors summarizes the current knowledge about these proteins, their structure, effects on nAChRs, and roles in cholinergic signaling (Miwa et al.). In a second publication on this topic, the first synthesis of water-soluble fragments of the central loop of Ly6 proteins and their binding to and effect on various nAChR subtypes are described, thereby identifying inhibition of the muscle type and therapeutically interesting α9α10 subtype by a Lynx1 fragment. Interestingly, the linear peptide and the disulfide bond-cyclized form had similar potency (Kryukova et al.). A third paper indirectly deals with three-finger folded proteins. Here, the soluble acetylcholine binding protein (AChBP), a valuable model for structural studies on nAChRs, was tested for its suitability as a potential treatment against snake bites. The authors describe efficient binding to and partial neutralization of long chain neurotoxins by an AChBP analog (Albulescu et al.).

Among the most selective tools in nAChRs research are α-conotoxins, comparably small peptides derived from the venom of marine predatory snails, and three papers in this issue are devoted to them. While several cryo-EM and X-ray structures of nicotine-bound nAChR exist, no full-length nAChR structure bound to a peptide or protein neurotoxin is known. Therefore, co-crystal structures of the AChBP or the ligand binding domains (LBD) of nAChRs provide important models. The paper by Zouridakis et al. shows for the first time the X-ray structure of an α-conotoxin, the α9α10-selective RgIA, in complex with the LBD of the α9 nAChR. Despite the monomeric state of the α9 LBD, computer modeling enabled the construction of a pentameric α9α10 model and determination of the RgIA binding subunit interface in agreement with published functional data. The α9α10-selective conotoxin RgIA has previously been shown to have anti-inflammatory and anti-nociceptive action in neuropathic pain models. However, the exact physiological mechanisms are incompletely understood (Dutertre et al., 2017). The review by Grau et al. describes the development of α7 and human α9α10-selective analogs of conotoxins ArIB and RgIA, respectively, and their use in the characterization of nAChRs in immune cells. They summarize data that show that theses receptors via a metabotropic mechanism involving the purinergic P2X7 receptor modulate inflammation. Finally, in the paper by El Hamdaoui et al., a strategy for the production of α-conotoxin TxIA variants in *E. coli* was tested. Although it was demonstrated that this resulted predominantly in the so-called “ribbon” isomer in which disulfide bonds are misfolded (compared to the naturally occurring “globular” α-conotoxins), a weak antagonistic potency of these peptides was determined, and a binding mode at the α7 nAChR was proposed. Nevertheless, the study showed that careful structural analysis is a requirement if disulfide bond formation cannot be controlled.

Three papers were devoted to spider toxins targeting voltage-gated Na^+^ and K^+^ channels. The review by Cardoso and Lewis summarizes the current understanding of the structure-activity relationships of spider cysteine knot peptides, which are a highly diverse and chemically stable group of peptides that allosterically modulate voltage-gated sodium channels (Na_V_) and have therapeutic potential for the treatment of pain. The study by Zhang Yunxiao et al. describes the identification and characterization of μ-TRTX-Ca2a, another spider toxin with activity at the Nav1.7 Na^+^ channel subtype and analgesic properties. Another group (Zhang Yiya et al.) performed an alanine scan to determine the molecular mode of action of Jingzhaotoxin-V at the Kv4.2 potassium channels in rat cardiomyocytes.

Finally, Dastpeyman et al. investigated the structure, stability, and activity of chlorotoxin fragments to identify regions that determine its functions. This scorpion toxin binds selectively to tumor cells and has been shown to inhibit cell migration. While the target of the toxin is not known, two C-terminal fragments appeared to retain some activity in cellular assays for surface binding, internalization, and migration.

Taken together, this Research Topic provides topical examples how toxins have been modified to identify the principal determinants of their action or to develop improved tools for ion channel research.

## Author Contributions

VT was invited by Frontiers in Pharmacology, suggested the title of this special issue and invited AN, CU, and J-FR as co-editors. The Editorial was written by AN and VT with comments from CU and J-FR.

## Conflict of Interest

The authors declare that the research was conducted in the absence of any commercial or financial relationships that could be construed as a potential conflict of interest.
